# Rare encounter: Osteoid osteoma of the coracoid process base – A case report and in-depth literature review

**DOI:** 10.1016/j.ijscr.2025.110826

**Published:** 2025-01-04

**Authors:** Ramin Zargarbashi, Maryam Salimi, Ghaffar Habibi Shekardasht, Maryam Sotoudeh Anvari, Seyedarad Mosalamiaghili

**Affiliations:** a Pediatric Orthopaedic Surgery, Children's Medical Center, Tehran University of Medical Science, Tehran, Iran; b Bone and Joint Diseases Research Center, Department of Orthopedic Surgery, Shiraz University of Medical Sciences, Iran; c Golestan Rhematology Research Center, Golestan University of Medical Sciences, Gorgan, Iran

**Keywords:** Osteoid osteoma, Shoulder, Coracoid, Case report

## Abstract

**Introduction and importance:**

Osteoid osteoma (OO) is a common benign bone tumor, mostly affecting young adults. Since it often develops in long bones, OO is rarely considered as a cause of chronic shoulder pain.

**Case presentation:**

We treated an 8-year-old boy with ongoing shoulder pain that was worse at night but improved with NSAIDs. Based on his symptoms, MRI and CT scans pointed to OO in the coracoid process. The tumor was successfully removed through open surgery, leading to immediate relief, and the boy remained pain-free in 24 months after the procedure.

**Clinical discussion:**

Osteoid osteoma in the coracoid process is rare, often causing diagnostic delays. Advanced imaging like CT and MRI is crucial for accurate diagnosis. In this case, open surgery ensured complete nidus removal due to neurovascular proximity, aligning with reported favorable outcomes.

**Conclusion:**

This case emphasizes the importance of considering rare causes like coracoid OO in shoulder pain, utilizing advanced imaging and tailored surgical approaches for successful outcomes.

## Highlights

• Osteoid osteoma is rarely considered as a cause of chronic shoulder pain.
• An 8-year-old boy presented with ongoing shoulder pain, worse at night but improved with NSAIDs.
• MRI and CT scans revealed osteoid osteoma in the coracoid process.
• After MRI and CT scan revealed osteoid osteoma; tumor was successfully removed through open surgery, leading to immediate relief.
• Early use of advanced imaging techniques is crucial for accurate diagnosis.
• Surgical excision can lead to excellent outcomes in young patients with chronic shoulder pain.

## 1 Background

Osteoid osteoma (OO) was first described by Jaffe in 1935 and is usually found as a benign bone lesion in the shafts of long bones [1]. It's very rare in flat bones, with only about 1 % of cases affecting the scapula [2]. Even less common is its occurrence in the coracoid process, with only a few reports in the literature. These cases often present with shoulder pain, and patients are sometimes treated for long periods with incorrect diagnoses, such as cervical radiculopathy or “periarthritis,” causing delays in reaching the right diagnosis [[3], [4], [5], [6], [7], [8], [9], [10], [11], [12], [13], [14]]. Treatments for these lesions range from more invasive surgical removal to minimally invasive procedures like percutaneous radiofrequency ablation [[3], [4], [5], [6], [7], [8], [9], [10], [11], [12], [13], [14]]. In this report, we present a case of OO in the coracoid process that was successfully treated with open surgery.

## 2 Case presentation

This work has been reported in line with the SCARE criteria [15]. An 8-year-old boy with no prior medical history came in with worsening shoulder pain over the past year. The pain, which was worse at night but improved with NSAIDs, did not radiate. He had no issues with nerve or blood vessel function, and no movement problems. There was also no history of trauma or musculoskeletal disorders in the patient or his family.

**Fig. 1:**
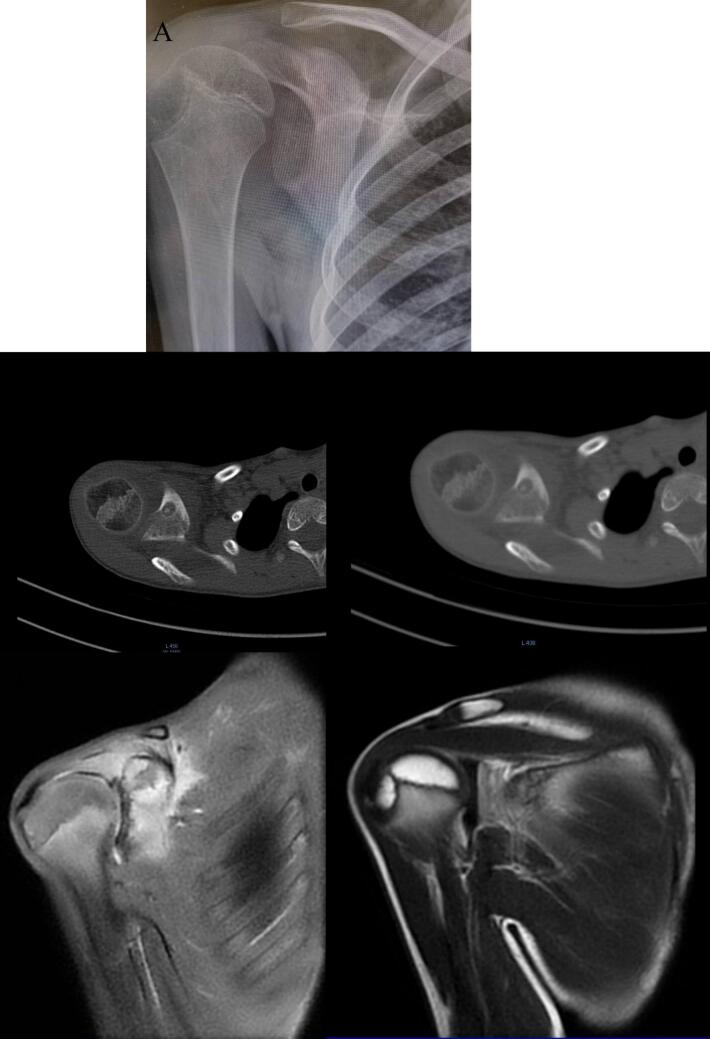
A: Thickening of the coracoid and scapula. B: Bone marrow swelling in the coracoid, along with increased bone thickness in the front of the scapula. There was also tissue swelling and fluid in the shoulder joint. C: A typical appearance of an osteoid osteoma (nidus) in the coracoid, with thickening of the bone cortex and surrounding tissues.

During the exam, a mild swelling was noted in the front of the shoulder and clavicle area. Initial X-rays showed thickening of the coracoid and scapula (Fig. 1A). An MRI revealed significant bone changes and swelling (Fig. 1B), and a CT scan showed a lesion on the base of the coracoid, suspected to be an osteoid osteoma (Fig. 1C).

### 2.1 Surgical procedure

**Fig. 2:**
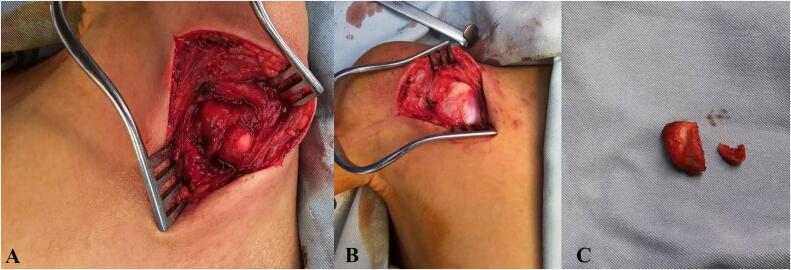
Figures showing the (A) dissection region, (B) Nidus being completely isolated and separated, (C) Removed Tissue of the Osteoid Osteoma.

An anterior deltopectoral approach was used to access the shoulder. The dissection was carried out between the deltoid and pectoralis major muscles to expose the coracoid. The pectoralis minor muscle was detached, at the level of medial coracoid, as were the tendons of the coracobrachialis and short head of the biceps from the lower part of the coracoid. The coracohumeral and coracoacromial ligaments were also detached from the lateral coracoid level. At this point, the coracoid was fully separated from the surrounding muscle. The neurovascular bundle, including the brachial plexus, was carefully protected under the pectoralis minor. The surgeon performed an ostectomy at the base of the coracoid, where the lesion (nidus) was located and completely removed (Fig. 2). The tissue was sent to pathology for further analysis. The complete removal of the coracoid left the muscle stump of the coracobrachialis attached to the pectoralis minor, but unattached elsewhere due to the coracoid's absence. The long head of the biceps, anchored to the glenoid process, prevents retraction, while the unattached short head of the biceps does not pose any functional issues.

### 2.2 Pathology results

**Fig. 3:**
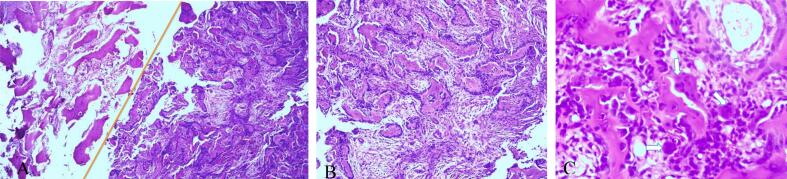
(A) Sharp border (orange line) of nidus and surrounding bone: Thickened trabeculae of lamellar bone (left) and trabeculae of woven bone with prominent osteoblastic rimming (right) (4×) (B) Nidus consists woven bone trabeculae of variable thickness lined by prominent osteoblastic rimming and intervening fibrovascular stroma (10×). (C) High power view of variable mineralized woven bone trabeculae and a single layer of plump osteoblastic rim. Scattered giant cells (arrows) are present within fibrovascular stroma (40×).

The analysis confirmed the lesion was a benign bone-forming tumor. It consisted of irregular trabeculae of immature bone (woven bone) with a rich blood supply and occasional osteoclasts. Additionally, thicker lamellar bone was found along with loose fibrovascular tissue (Fig. 3).

### 2.3 Follow-up and future care

**Fig. 4:**
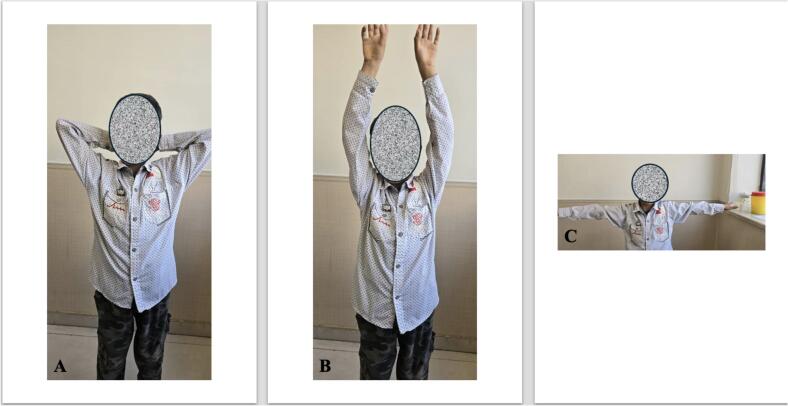
Pain free complete range of motion in last follow-up.

**Fig. 5:**
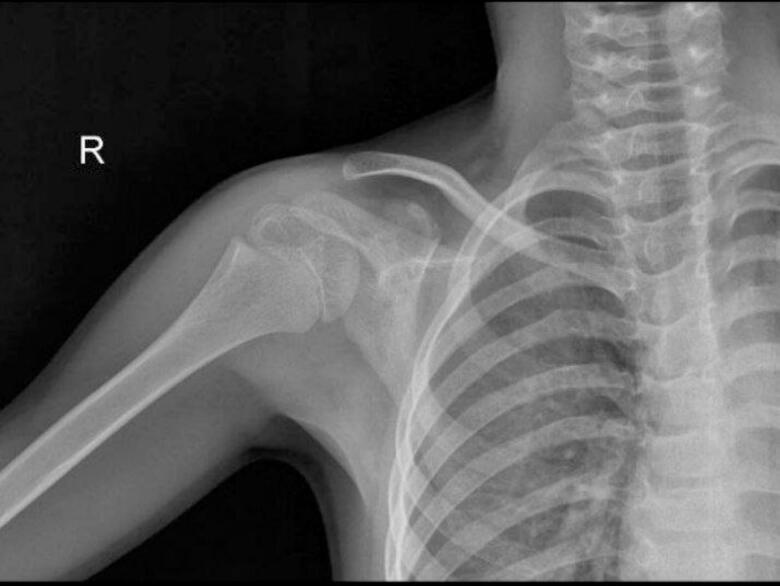
Post-op X-ray showing the result of the healing after 24 months.

After surgery, the patient wore a sling for 10 days. Physical therapy and range of motion exercises started after that. At follow-up visits at 1, 3, 6, 12, and 24 months, the patient had no pain or recurrence of the condition and no reported limited range of motion was detected. (Fig. 4, Fig. 5).

## 3 Discussion

Osteoid osteoma of the coracoid process is an exceptionally rare occurrence, with only a handful of cases reported in the literature [2]. The scarcity of coracoid process OO cases often leads to misdiagnosis and delayed treatment, as seen in our patient's year-long history of worsening pain. The more likely diseases of the shoulder pain, causes the benign tumor to rarely fall into consideration [8]. Table 1 Shows the literature summary of shoulder OO.

**Table 1:** literature summary of shoulder osteoid osteoma.

	Author year	Patient info	Location in shoulder	Sign and symptoms	Radiological features	Management/surgical process	Reason for choosing the procedure	Diagnosis delay
1	Rouhani et al., 2017 [17]	25 M	Neck of left scapula	Night pain, radiating arm	X-ray: non-specific sclerosis; MRI: high-signal area in T2-weighted images with a low-signal ring; CT: small calcification and low-intensity nidus; Bone scan: Tc99 detected focal lesion.	Surgical resection via deltopectoral approach; tenotomy of upper subscapularis; complete tumor removal.	Surgical resection chosen due to inaccessibility of the tumor by routine approaches and necessity to fully expose coracoid base and glenoid neck for complete excision	27 months
2	Kelly et al., 2002 [9]	30 M	Anteroinferior aspect of the right glenoid	Impingement sign, night pain	MRI: labral tear; narrow subacromial space; Bone scan: focal signal increase in anteroinferior glenoid; CT: nidus in glenoid cortex.	Arthroscopy: initial acromioplasty and SLAP lesion repair followed by arthroscopic excision of the osteoid osteoma.	Persistent pain after previous interventions, bone scan findings suggestive of osteoid osteoma	2–3 years
3	Kelly et al., 2002 [9]	12 M	Base of the coracoid of the right shoulder	Night pain, weakness	MRI: nidus (5 mm) at the base of coracoid with marrow edema and joint effusion; CT confirmed typical nidus with surrounding sclerosis.	Arthroscopic excision of the nidus with complete removal.	History, clinical presentation, and MRI findings indicative of osteoid osteoma	6 months
4	Akpinar et al., 2001 [3]	14 F	Base of coracoid process	Limited motion, night pain	X-ray: no abnormalities; CT: nidus in subcortical region with marrow edema; MRI: hypointense nidus and extensive bone marrow edema.	Open surgery: en bloc excision; coracoid osteotomy with wide marginal excision and reattachment with malleolar screw.	Surgical excision was chosen due to the rarity of the lesion and to ensure complete removal by accessing the tumor via an anterior approach, followed by secure fixation with a screw.	4 years
5	AlGhoozi et al., 2020 [21]	22 M	Base of coracoid process	Night pain, no NSAID relief	MRI: suspicious lesion in coracoid; CT: benign osteoid osteoma with reactive sclerosis.	Arthroscopic excision with image intensification and debridement; oral analgesics postoperatively.	Arthroscopy was chosen due to its minimally invasive nature, guided by imaging, resulting in a quicker recovery and complete resolution of symptoms	2 years
6	Babaei et al., 2024 [19]	13 M	Base of coracoid process	Radiating pain, partial NSAID relief	X-ray: normal findings; MRI: marrow edema in scapula, glenoid, and coracoid; CT: nidus with cortical thickening and reactive sclerosis.	Radiofrequency ablation (RFA): CT-guided thermal ablation at 90¬∞C for 7 min, avoiding brachial plexus.	Minimally invasive, precise targeting of nidus with low risk to vital structures (e.g., brachial plexus) and quicker recovery compared to open surgery	1 year
7	Babaei et al., 2024 [19]	20 M	Base of coracoid process	Night pain, limited motion	MRI: reactive sclerosis and cortical thickening; CT: nidus confirmed at coracoid base.	RFA: similar CT-guided thermal ablation to the first case; precise targeting to avoid neurovascular damage.	Minimally invasive, effective for treating OO, providing accurate destruction of the nidus while reducing risks to adjacent anatomical structures	1 year
8	Paragjyoti Gogoi et al., 2013 [16]	12 M	Base of coracoid process	Dull pain, tenderness	X-ray: radiolucent lesion at base of coracoid with surrounding sclerotic margin; CT: 4 mm lesion with no intra-articular pathology.	De-roofing and curettage via anterior deltopectoral approach.	Familiarity with delto-pectoral approach, relatively superficial location of the lesion	4 months
9	Dorotea Božić et al., 2016 [18]	22 M	Coracoid process (left)	Night pain, constant	MRI: cystic lesion in coracoid with periosteal reaction; CT: nidus with mild surrounding sclerosis.	Open surgical excision of the nidus with intraoperative imaging confirmation.	Open surgery was chosen due to proximity to neurovascular structures, making RFA unsuitable; open approach preserves the functional role of the coracoid process	1 year
10	I. Degreef et al., 2005 [24]	53 F	Acromion (right shoulder)	Night pain, tenderness	X-ray: mild osteolysis of the acromion; CT: radiolucent zone; Bone scan: hypercaptation in the acromioclavicular joint.	Open resection of the acromion; immediate post-surgical pain relief.	Open surgery allowed for diagnosis confirmation and ensured complete removal of osteoma	6 months
11	Łukasz Chojecki et al., 2017 [25]	14 F	Acromion	Shoulder pain (omalagia)	Lesion diagnosed as OO; Imaging findings not specified.	Arthroscopic removal of the lesion.	Arthroscopic technique was chosen for less invasive access and effective symptom relief	NR
12	Michael C. Glanzmann et al., 2011 [4]	22 M	Coracoid process	Night pain, restricted motion	MRI: bone marrow edema and periosteal reaction; CT: dense nidus with reactive bone.	Arthroscopic excision with capsular release.	Arthroscopy allowed for full access to the coracoid process and treatment of both the tumor and the associated stiffness in one procedure	18 months
13	Goyal et al. 2015 [5]	27 M	Coracoid process	Night pain, scapular dyskinesia	MRI: nidus surrounded by marrow edema and joint effusion; CT: nidus confirmed in coracoid base.	Arthroscopic excision with debridement and nidus removal.	Arthroscopic excision allowed direct visualization, less morbidity than open surgery, minimal risk to neurovascular structures	2 years
14	Amin et al., 2020 [26]	18 F	Scapular glenoid	Night pain, sweating	MRI: high T2, low T1 signal; CT: well-defined central nidus with cortical thickening.	CT-guided RFA with complete nidus ablation.	Non-invasive, good results, quicker recovery	5 years
15	Miyazaki et al., 2014 [27]	46 F	Acromion	Night pain, physical activity worsens	MRI: severe inflammation and cyst; CT: nidus with cystic changes and sclerosis.	Arthroscopic resection of nidus with acromioplasty (Mumford procedure).	To achieve complete removal of the lesion with minimal tissue damage	3 months
16	Hideki Ueyama et al. 2015 [28]	16 M	Base of the coracoid process	Night pain, restricted motion	MRI: tumor with significant bone marrow edema; CT: nidus with surrounding sclerosis and cortical changes.	Arthroscopy with curettage and radiofrequency ablation.	To address the lesion at the base of the coracoid process, which was difficult to access via open surgery; to treat accompanying intra-articular lesions like synovitis	7 months
17	Kamrani et al. 2017 [29]	Mean age: 26 ± 9 years, All male (M)	N/A (focuses on elbow)	Significant pain (VAS: 79 ¬ ± 7 pre-op, 3 ¬ ± 2.1 post-op), responsive to NSAIDs	CT: nidus size 8.7 ¬ ± 3.7 mm; reactive sclerosis	Arthroscopic ablation; revision surgeries for unsuccessful cases (including open surgical release)	Aimed to evaluate medium-term functional effects of arthroscopic ablation (considered safe and efficient)	Mean consultation: 3 months; Diagnosis delay: 23.4 months

R = right; L = left; NR = not reported; RFA = radiofrequency ablation; ROM = range of motion; CT = computed tomography; MRI = magnetic resonance imaging; OO = osteoid osteoma; NSAIDs = non-steroidal anti-inflammatory drugs; VAS = visual analog scale; SLAP = superior labrum anterior to posterior (lesion).

Diagnostic challenges and imaging considerations are paramount in such cases. As noted in the literature, initial X-rays often fail to reveal abnormalities in coracoid process OO [16]. This was evident in our case, where initial radiographs only showed thickening of the coracoid and scapula. The subsequent use of MRI and CT scans proved crucial in accurately identifying the lesion [3]. This aligns with Ogose et al.'s recommendation for early use of advanced imaging modalities like CT or MRI to visualize the characteristic nidus more clearly [14].

The differential diagnosis for chronic shoulder pain in young patients is broad, and OO is rarely considered initially. Previous cases have been misdiagnosed as impingement syndrome [9], arthritis [3], or cervical spine discopathy [17]. Notably, at 8 years old, our patient is younger than the typical age range (12–46 years) [5] reported in most cases also his symptoms of night pain and relief with NSAIDs are typical of OO, yet the unusual location led to a prolonged diagnostic process.

Treatment options for coracoid process OO range from open surgical excision to minimally invasive techniques [18]. While our case was managed with open surgical excision, it's worth noting that percutaneous radiofrequency ablation (RFA) and arthroscopic approaches have been successfully employed in some cases [19,20]. The choice of treatment modality depends on various factors, including the exact location of the lesion, proximity to neurovascular structures, and available expertise [21]. The anatomical considerations of the coracoid process location present unique challenges. The proximity to important neurovascular structures, like musculocutaneous nerve underneath the coracobrachial muscle, makes techniques like RFA potentially risky [18]. In our case, open surgery allowed for complete removal of the nidus, leading to immediate pain relief and excellent long-term outcomes. In surgical procedures, complete excision of the core is generally the preferred method. Campanacci's two proposed techniques involve either wide excision of the entire area or gradual removal of the overlying bone, followed by thorough scraping. For lesions in critical areas, it is suggested to unroof and perform curettage. In 2002, Kelly first reported using arthroscopy as a treatment method for OO in the shoulder [9]. Arthroscopic treatment of OO in upper extremity joints has shown high success rates with few complications, though there remains a risk of incomplete removal in hard-to-reach areas during the procedure [22].

Pathology results confirmed our diagnosis, showing irregular trabeculae of immature woven bone with rich vascularity, consistent with typical OO histology. Long-term outcomes for patients with coracoid process OO after surgical excision are generally favorable [21]. Our patient remained pain-free at 24 months post-surgery, which is consistent with the literature reporting quick return to daily activities and symptom-free follow-ups [19]. Although osteoid osteomas are benign bone tumors that rarely exhibit aggressive behavior or potential for malignant transformation, there are exceedingly rare cases documented in the literature where OO has progressed to high-grade osteosarcoma. In this case, there was no evidence of malignant progression. The patient responded well to surgical excision, with no recurrence or complications observed during the 24-month postoperative period. Nevertheless, clinicians should remain vigilant, with regular follow-up imaging to monitor for any unexpected changes, even though the risk of malignancy is minimal [23].

This case report highlights the need to consider rare bone tumors, such as OO, in the differential diagnosis of chronic shoulder pain, particularly in pediatric patients. Advanced imaging modalities like CT and MRI are essential for timely and accurate diagnosis. The case also underscores the challenges of treating lesions in anatomically complex areas, emphasizing the importance of tailored surgical techniques based on lesion location and expertise.

Finally, this report underscores the importance of long-term follow-up to ensure symptom resolution and monitor for potential complications, including recurrence or malignant transformation. By combining thorough imaging, accurate diagnosis, and appropriate surgical intervention, favorable outcomes can be achieved in these rare cases.

## CRediT authorship contribution statement

All authors contributed to the study's conception and design. Material preparation and data collection were performed by RZ and GSH and MSA. The first draft of the manuscript was written by MS, and SM, and all authors commented on previous versions of the manuscript. All authors read and approved the final manuscript.

## Consent to participate

Written informed consent was obtained from the patient's parents/legal guardian for publication and any accompanying images. A copy of the written consent is available for review by the Editor-in-Chief of this journal on request.

## Ethics statement

The ethical committee of Tehran University of Medical Sciences approval was not required; the reason being the article type (case report).

## Guarantor

The Guarantor is the one or more people who accept full responsibility for the work and/or the conduct of the study, had access to the data, and controlled the decision to publish.

Seyedarad Mosalamiaghili, MD, Golestan University of Medical Sciences, Gorgan 49138-15739, Iran.

E-mail address: aradmosalami@gmail.com

## Funding sources

The authors declare that no funds, grants, or other support were received during the preparation of this manuscript.

## Registration of research studies

1. Name of the registry: Not Applicable.

2. Unique identifying number or registration ID: Not Applicable.

3. Hyperlink to your specific registration (must be publicly accessible and will be checked):

Not Applicable.

## Declaration of competing interest

None declared.
